# Impurities in Commercial Samples of Peroxide of Hydrogen

**Published:** 1892-02

**Authors:** 


### Impurities in Commercial Samples of Peroxide of Hydrogen.

In the *Medical News* of January 30, Dr. Samuel S. Wallian, of New York, demonstrates conclusively that the various samples of this agent, now in the market, vary greatly in their purity and efficiency. Of five samples one gave a strength of eight and one-quarter volumes, another of thirteen and a half volumes, a third of seventeen volumes, a fourth of one and one-half volumes and a fifth of twelve volumes. Some were distinctly acid in reaction and showed a considerable liberation of gas, in the force with which the cork of the bottle was ejected. The writer insists that samples should be entirely neutral in reaction, should not readily deteriorate, and should have the strength claimed for them. Only one of the samples tested answered these requirements.

A remedy which is so generally used as this, and in which purity is so important, should be known to be reliable, and we have for this reason called the attention of our readers to it.

